# Reproducibility of the three-dimensional endoscopic staging system for nasal polyposis

**DOI:** 10.1016/S1808-8694(15)30542-5

**Published:** 2015-10-19

**Authors:** Marcelo Castro Alves de Sousa, Helena Maria Gonçalves Becker, Celso Gonçalves Becker, Mariana Moreira de Castro, Nicodemos José Alves de Sousa, Roberto Eustáquio dos Santos Guimarães

**Affiliations:** 1 MSc - Medical School of the Federal University of Minas Gerais (FM-UFMG. Adjunct Professor - School of Medical Sciences of Minas Gerais - FCMMG; 2 PhD - FM-UFMG. Adjunct Professor - Department of Otorhinolaryngology and Ophthalmology - FM-UFMG; 3 PhD - FM-UFMG. Adjunct Professor - Department of Otorhinolaryngology and Ophthalmology - FM-UFMG; 4 Third year resident of Otorhinolaryngology -UFMG; 5 Full Professor of Otorhinolaryngology - FCMMG. Head of otorhinolaryngology - Santa Casa BH; 6 Associate Professor - USP - Ribeirão Preto. Adjunct Professor of Otorhinolaryngology - Department of Otorhinolaryngology and Ophthalmology - FM-UFMG

**Keywords:** neoplasm staging, nasal polyps, endoscopy, steroids

## Abstract

Nasal Polyposis is a chronic inflammatory process of the nasal mucosa, characterized by multiple and bilateral nasal polyps. Different drugs have been used in its treatment. In order to study the results of different treatment modalities it is necessary to have some kind of staging.

**Aim:**

to present a new endoscopic staging method, based on nasal endoscopy and on the three-dimensional nasal polyp assessment; and compare its reproducibility with that from two other systems already established in the literature.

**Study design:**

Cohort study.

**Material and methods:**

Three experts assessed the exams of 20 patients with nasal polyposis at different times, before, at 15 and at 30 days after the start of oral prednisone, 1 mg/kg/day, during 7 days. We assessed the agreement rate among the experts, using Kappa for statistic analysis.

**Results:**

The three methods were reproducible, and the method hereby proposed had the least agreement among the examiners.

**Conclusions:**

the three-dimensional staging system proposed proved reproducible, despite showing less agreement among the examiners than the other as the other two methods.

## INTRODUCTION

Nasal polyposis (NP) is a chronic inflammatory process of the nasal mucosa, characterized by the presence of multiple and bilateral nasal polyps. Its pathophysiology is still unclear, with numerous theories described in the literature.

It is a clinical manifestation of diseases with different etiologies, such as: non-allergic eosinophilic rhinitis, asthma, aspirin intolerance, cystic fibrosis, Kartagener syndrome, Young and Churg-Strauss, among others. Its prevalence in the general population varies between 0.5 and 4 % according to some authors^1^. NP tends recurr^2^, which makes its treatment a challenge for ENTs.

NP treatment involves the use of different drugs, especially topical and systemic steroids and surgical procedures. Many literature papers already show the efficacy of steroids in its treatment^3^, ^4^, ^5^, ^6^. Treatment goal is to reduce polyp size or, if possible, eliminate them, with consequent symptom relief, especially of nasal obstruction, hyposmia and anosmia, as well as to reduce the frequency of infections and improve associated lower airway symptoms, besides preventing complications such as mucoceles and orbit involvement. Steroids are also indicated in the preparation of these patients for surgery. Surgery is reserved for cases of clinical treatment failure.

Some type of NP staging is recommended in order to follow disease evolution in these patients, just as for the comparison between different types of treatment. For NP staging it is necessary to use the endoscope.

The literature describes numerous ways of staging NP using nasal endoscopy and there is still no method of universal consensus^15^, ^16^, ^17^, ^18^, ^19^, ^20^. Most of them classify nasal polyps in a two dimensional way in the nasal cavities and in relation to the middle meatus and outside of it.

Studies have been carried out in an attempt to compare the agreement of different tomographic staging for Chronic Rhinosinusitis^7^, ^8^, ^9^, ^10^ and only one study compared endoscopic staging among different observers^6^. In our setting, Stamm proposed a staging system based on CT Scan.

The staging to be tested in the present study, proposed by one of the authors, is based only on nasal endoscopy and it is a tridimensional evaluation of the polyps, in three spatial planes: horizontal, vertical and antero-posterior. It has been used in the ENT department of the Medical School Hospital of the Federal University of Minas Gerais for many years and it is believed to be the one which bests represents the real extension of nasal polyposis, besides precisely telling the location of nasal polyps in the nasal cavities.

## OBJECTIVES

The goal of the present study was to show a new type of NP endoscopic staging system, assess its reproducibility among different examiners and compare it with the other two methods described in the literature (Lund-Mackay and Johansen)^12^,^13^.

## MATERIALS AND METHODS

We selected 20 patients from the ENT Department of Federal University of Minas Gerais Medical School, bearers of eosinophilic nasosinusal polyposis. All the patients were volunteers, were educated about the procedure and signed the free and informed consent form. This study was approved by the Ethics Committee of the institution where it was done, under protocol number ETIC 171-6.

We excluded patients with some contraindication to use systemic steroids, as well as diabetics or hypertensive patients without proper control of their diseases. We also excluded those patients previously submitted to nasal surgeries.

The patients were previously submitted to polyp biopsy for the diagnosis of eosinophilic polyposis and should be at least 30 days without the use of oral or systemic steroids. The biopsies were carried out with a cup forceps with the help of an endoscope in cases of small polyps, or anterior rhinoscopy in cases of large polyps.

After the biopsy result, the patients were submitted to three endoscopic exams in their nasal cavities. After the first exam, the patients were medicated with 1mg/kg/day of oral prednisone up to the maximum dose of 60 mg, for a period of seven days. The second exam was held on the 15th and the 30th days after the first.

Thus, we assessed a total of 20 patients with bilateral polyposis, at three moments, corresponding to a total of 120 nasal cavities studied.

The patients were previously submitted to anterior rhinoscopy with vasoconstriction of the inferior turbinate through a cotton ball soaked in naphazoline.

The endoscopies were always carried out by one single examiner, with a 4mm and 30° rigid endoscope and in cases of anatomical alterations, such as septal deviation, we also used the 3.2mm flexible nasal fiberscope. The exams were recorded in a VHS video system for further assessment.

The exam started in the right nasal cavity, inspecting its floor, all the way to the choana. Whenever possible, we visualized the spheno-ethmoidal recess, then the middle meatus and the superior region of the nasal cavities, trying to see the polyps in the three planes.

After doing all the exams, they were copied to a single DVD disk, of which copies were made and handed over to the other two examiners simultaneously.

### Staging

**Figure 1:**
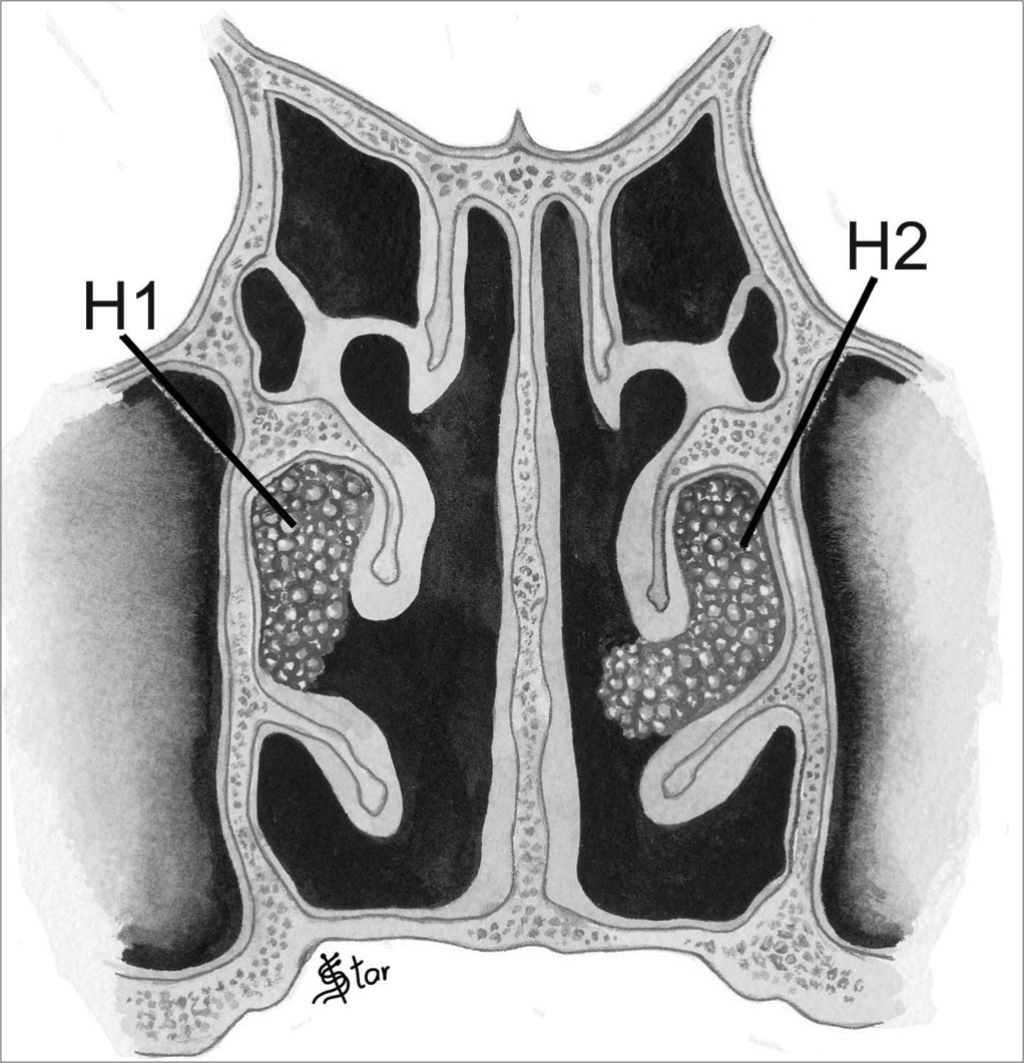
NP staging in the horizontal plane - H1 - polyps restricted to the middle meatus. H2 - Polyps getting out of the middle meatus without touching the septum

**Figure 2:**
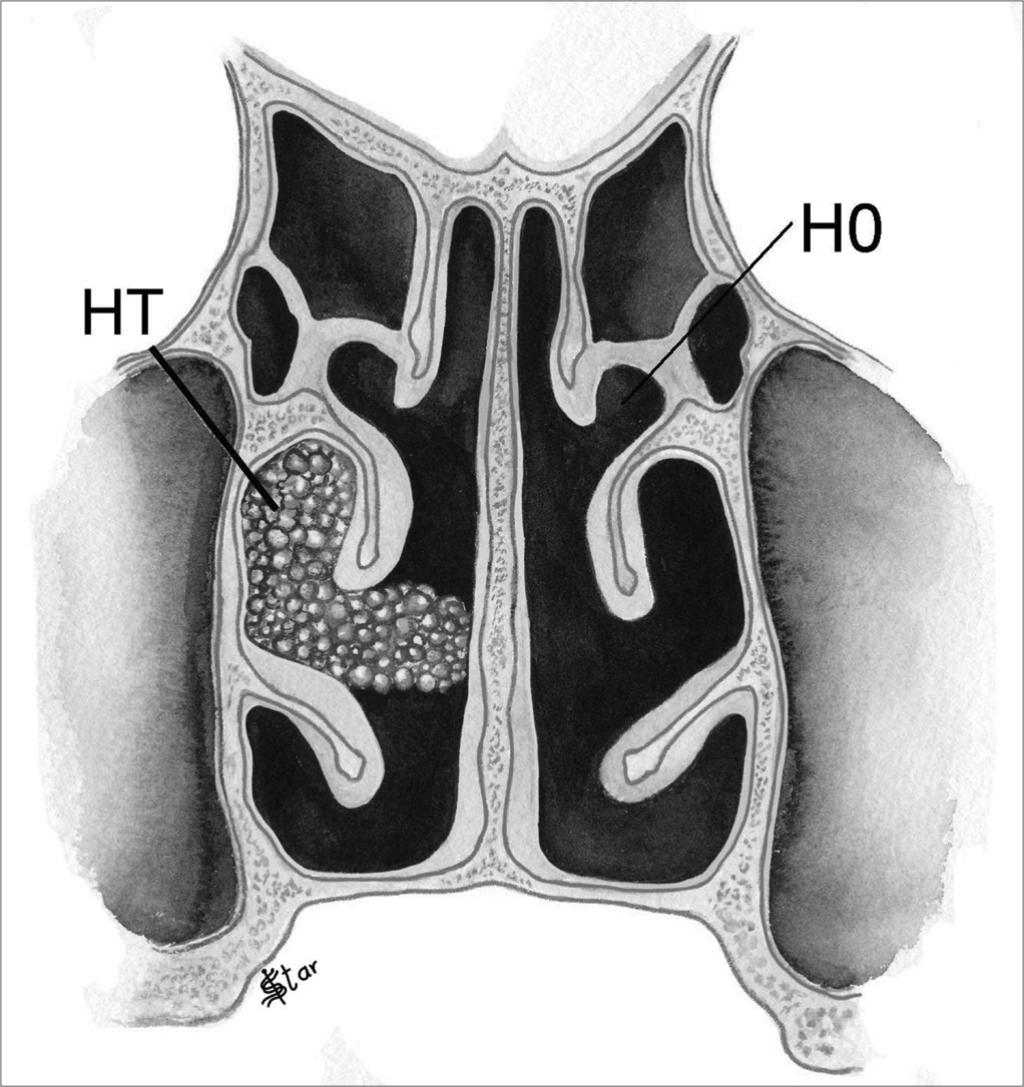
NP staging in the horizontal plane - H0 - No polyps. HT -Polyps extending beyond the middle meatus and touching the septum
  In the vertical plane (V), the polyps are classified as (Figure 3, Figure 4):
  – V0 - No polyps
  – V1 - Polyps in the middle meatus only
  – VI - Polyps extending inferiorly to the middle meatus, going beyond the upper border of the inferior turbinate
  – VS - Polyps extending superiorly to the middle meatus, between the septum and the middle turbinate
  – VT - Polyps occupying the entire vertical aspect of the nasal cavity

1) Tridimensional Staging:
  This staging provides information on the location of the polyps in the nasal cavity in the three dimensions of space, that is, in the antero-posterior, horizontal and vertical planes.
  In the horizontal plane (H), polyps were classified as (Figure 1, Figure 2):
  – H0 - No polyps
  – H1- Polyps restricted to the middle meatus
  – H2 - Polyps expand beyond the middle meatus, without touching the nasal septum.
  – HT - Polyps expand beyond the middle meatus and touch the septum.

**Figure 3:**
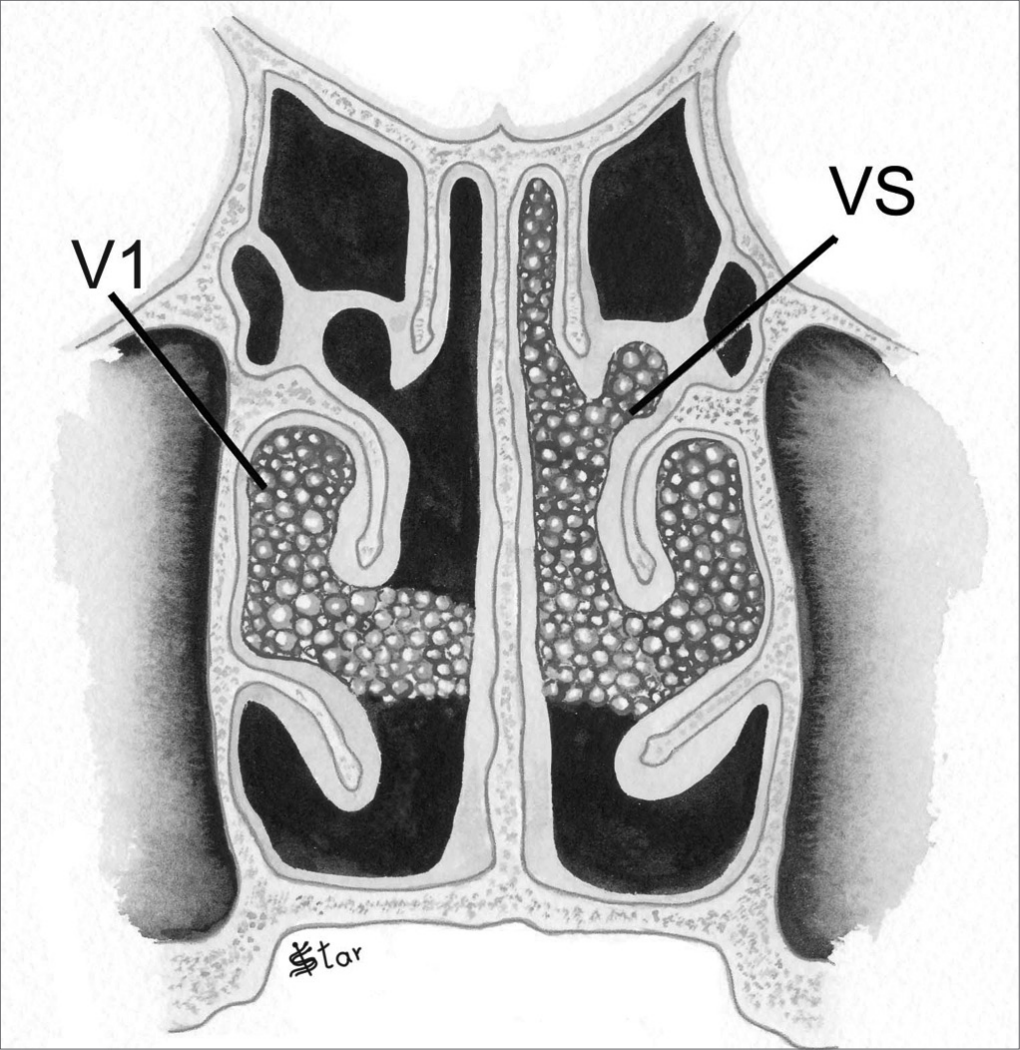
NP staging in the vertical plane - V1 - Polyps in the middle meatus only. VS - Polyps extending superiorly to the middle meatus

**Figure 4:**
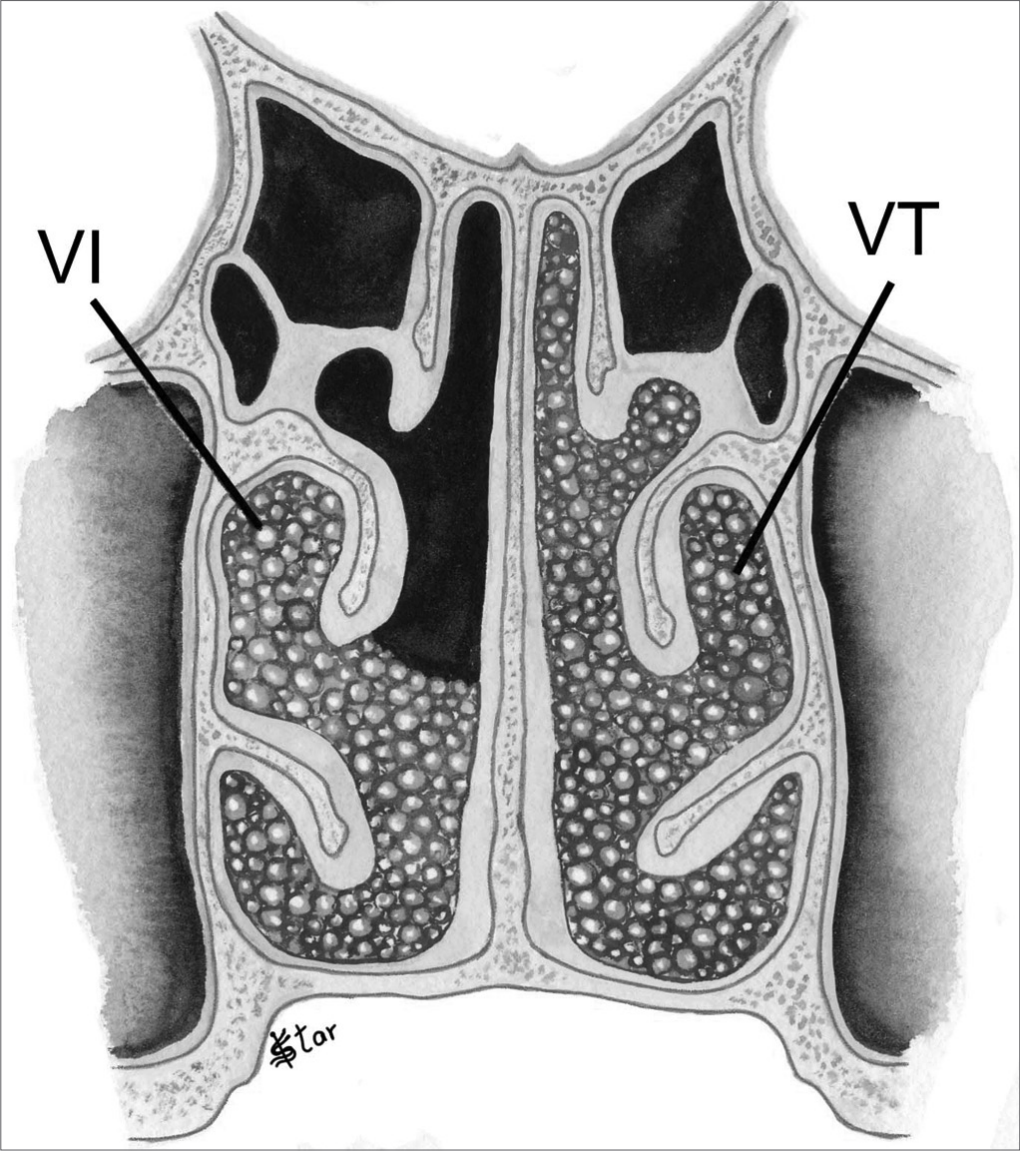
NP staging in the vertical plane - VI - Polyps extending inferiorly to the middle meatus. VT - Polyps occupying the entire vertical aspect of the nasal cavity
  In the antero-posterior plane (P), the polyps are classified as (Figure 5, Figure 6, Figure 7, Figure 8):
  – P0 - No polyps
  – P1 - Polyps in the middle meatus only
  – PA - Polyps extending anteriorly to the middle meatus, reaching the head of the inferior turbinate
  – PP - Polyps extending posterior to the middle meatus, reaching the tail of the inferior and middle turbinate
  – PT - Polyps occupying the entire antero-posterior aspect of the nasal cavity

**Figure 5:**
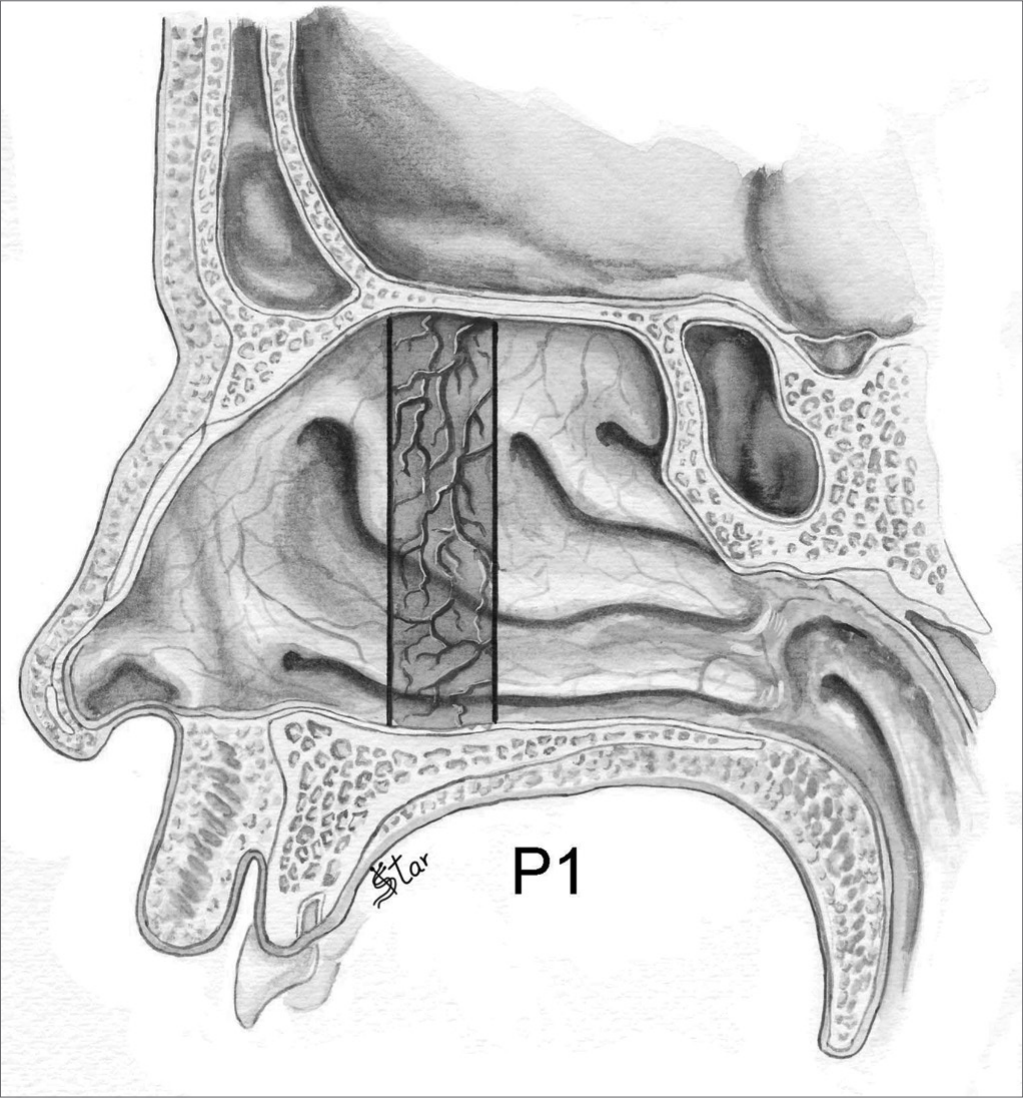
NP staging in the antero-posterior plane - P1 - Polyps in the middle meatus region only

**Figure 6:**
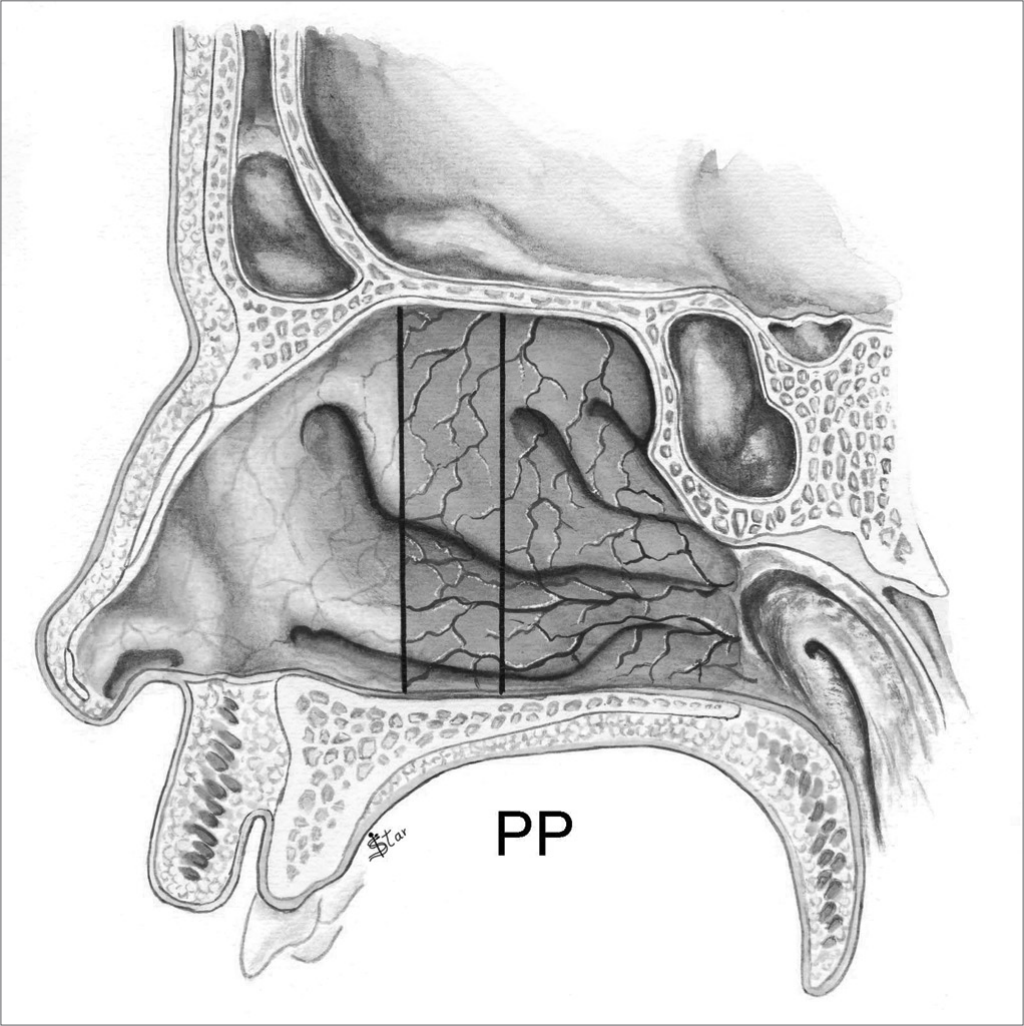
NP staging in the antero-posterior plane - PP - Polyps extending posterior to the middle meatus

**Figure 7:**
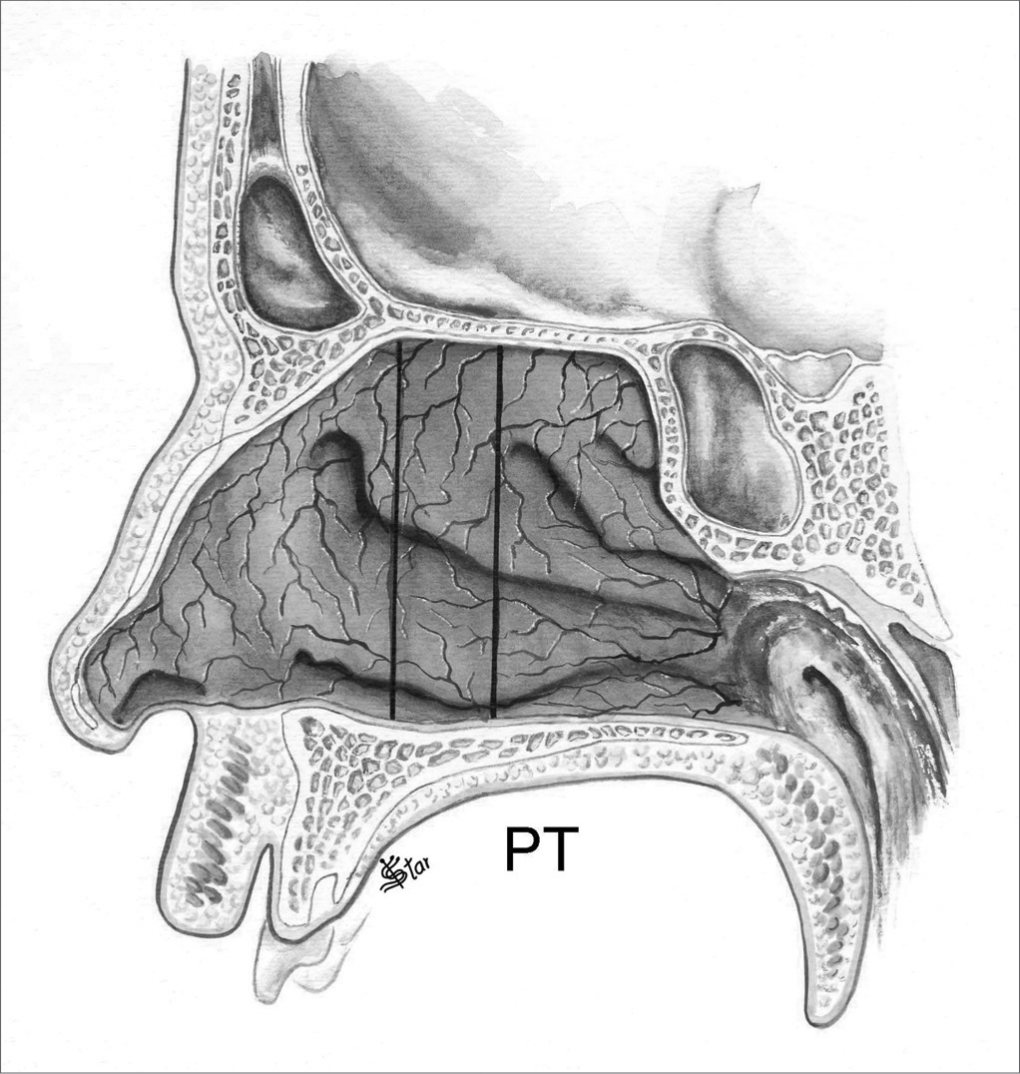
NP staging in the antero-posterior plane - PT - Polyps occupying the entire antero-posterior aspect of the nasal cavity

**Figure 8:**
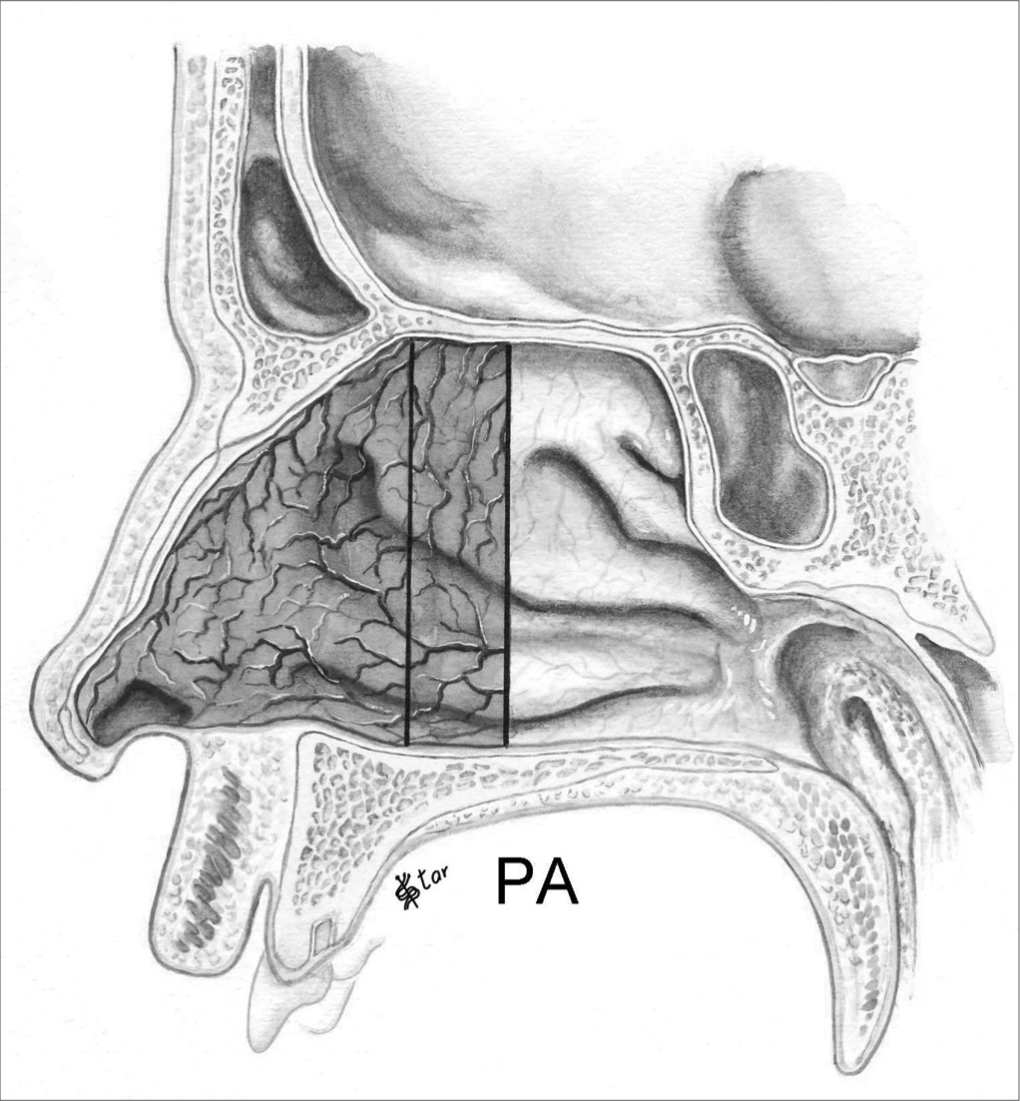
NP staging in the antero-posterior plane - PA - Polyps extending anteriorly to the middle meatus
2) Lund-Mackay^12^ Staging
  In this endoscopic evaluation, polyps are classified as:
  0- No polyps
  1 - Polyps in the middle meatus only
  2 - Polyps expand beyond the middle meatus
  3) Johanssen Staging^13^
  In this staging, the polyps are assessed in relation to the middle meatus and inferiorly on the following way:
  0 - No polyps
  1 - Polyps restricted to the middle meatus (Mild polyposis)
  2 - Polyps expand beyond the middle meatus, without going beyond the lower border of the inferior turbinate (moderate polyposis)
  3 - Polyps expand beyond the lower border of the inferior turbinate (Severe polyposis)

### Statistical Analysis

The three classifications were assessed for the right and left sides, by three examiners for the 20 patients.

In order to assess the degree of agreement among the examiners, we used the Multiple Kappa coefficient. Such coefficient can be interpreted as being a mean value of the agreement coefficients among examiners two by two. Based on sample values, as estimated by the Multiple Kappa, as well as its respective 95% confidence interval and we also calculated the p-value. When there was agreement among examiners, its level varied according to the classification below.

The classifications of the calculated coefficients correspond to the ones presented on Table 1:

**Table 1:** Kappa assessment

Kappa	Evaluation
< 0,20	Poor

0,21 a 0,40	Reasonable
0,41 a 0,60	Moderate
0,61 a 0,80	Good
0,80 a 1,00	Very good

The statistical analyses were carried out using the software R - of public domain, and the conclusions were drawn from results obtained considering a 5% level of significance.

## RESULTS

The estimates of the Kappa coefficients and the respective confidence intervals are presented on Table 2. We notice that for the evaluation of the right side, the Lund-Mackay method did not present significant agreement among the examiners at moments 1 and 3 and, regarding the horizontal tridimensional method, the agreement was not significant at moment 3.

**Table 2:** Kappa coefficient for the agreement among the examiners.

Side	Moment			Classifications		
		TRI(H)	TRI(V)	TRI(P)	Lund-Mackay	Johansen

	1	0,84 (0,35 a 1,00)	0,33 (0,15 a 0,51)	0,35 (0,17 a 0,53)	0,45*(0,00 a 1,00)	0,65 (0,42 a 0,89)
Right	2	0,56 (0,27 a 0,85)	0,37 (0,17 a 0,58)	0,42 (0,21 a 0,63)	0,68 (0,24 a 1,00)	0,64 (0,41 a 0,88)
	3	0,37*(0,00 a 0,86)	0,32 (0,10 a 0,53)	0,28 (0,08 a 0,48)	0,44*(0,00 a 1,00)	0,56 (0,29 a 0,83)
	1	0,83 (0,42 a 1,24)	0,49 (0,31 a 0,67)	0,57 (0,39 a 0,74)	0,80 (0,34 a 1,00)	0,78 (0,54 a 1,00)
Left	2	0,75 (0,47 a 1,00)	0,39 (0,18 a 0,59)	0,58 (0,38 a 0,79)	0,71(0,37 a 1,00)	0,74 (0,52 a 0,96)
		0,87 (0,57 a 1,00)	0,54 (0,39 a 0,70)	0,51 (0,33 a 0,68)	0,84 (0,45 a 1,00)	0,90 (0,69 a 1,10)

Legend: * p value ≫ 0.05; (): 95% confidence interval.

All the other evaluations of the right side and all those carried out on the left side had significant agreement levels (p value ⩽ 0.05). The best agreements (good and very good) were found for the horizontal tridimensional method, Lund-Mackay and Johansen on the left side and at the three assessment times.

Table 3 shows the translation of the results obtained on Table 1 by the classification of the evaluations of the agreement levels among the examiners.

**Table 3:** Evaluation of the agreement among the observers.

Side	Moment			Classifications		
		TRI(H)	TRI(V)	TRI(P)	Lund-Mackay	Johanssen
	1	Very good	Reasonable	Reasonable	–	Good
Right	2	Moderate	Reasonable	Moderate	Good	Good
	3	–	Reasonable	Reasonable	–	Moderate
	1	Very good	Moderate	Moderate	Very good	Good
Left	2	Good	Reasonable	Moderate	Good	Good
	3	Very good	Moderate	Moderate	Very good	Very good

Legend: -: Agreement without statistical agreement.

## DISCUSSION

NP staging is very important to assess response to different types of treatment - especially clinical, and to compare treatment results among different authors. It must be carried out using nasal endoscopy preferably and, if possible, also CT scan. In some cases, the polyps are only diagnosed with the endoscope. It is also important to cause a vasoconstriction to the lower turbinated prior to the exam in order to facilitate the view of the nasal cavities and polyps. In a paper about endoscopic staging, Johansson et al. suggested that the endoscopies should be carried out without the prior use of decongestants, under the risk of them reducing polyp size^10^. Later on, they studied the effects of topical decongestants on the polyps and did not notice any effect^14^.

In order to validate some type of staging, it must be reproducible among different examiners and easy to interpret and perform in the clinical practice. It is also important that the method be sensitive enough to detect small changes in polyp size.

Some types of endoscopic staging were proposed by different authors. Levine^15^, Jorgensen^16^, Mackay & Nacleiro^17^, Rasp et al.^18^, Passali et al^19^, Meltzer et al^20^ & Lund^3^ classified polyps in three of four categories, with some variations according to their location in the middle meatus and beyond it, and somehow related to the middle and lower turbinates, in most of the cases.

All these staging methods, including the ones used in the present paper, basically evaluate polyps in relation to the middle meatus or with inferior extension in most of the cases. One of them also assesses the upper region^18^. The tridimensional staging proposed in this paper has the advantage of informing polyp location in the three spatial planes and classifying polyps in other regions which are not the middle meatus. By classifying the polyps in the three dimensions, the specialist has a more accurate idea of the polyp's extension and location, without the need to see the exam. Contrary to this, the other methods do not provide this visualization. For example, a single and extensive polyp extending beyond the middle meatus inferiorly all the way to the nasal cavity floor would be classified as Johansen 3 and Lund-Mackay 2, which correspond to the maximum degree of polyposis extension. In the case of tridimensional staging, it would be HT VI P1. And in the case of extensive polyposis, it would be HT VT PT.

There is only one paper in the literature which assessed the reproducibility of endoscopic staging in NP and compared them11. This study used the same two staging systems reported in the literature compared here, and one assessment based on a computerized program. The results were similar to the ones found in relation to the Johanssen's and Lund-Mackay systems, which proved to be reproducible, and the former presented better agreement.

In the tridimensional staging, the vertical and antero-posterior planes showed the worst agreement. In cases of high septal deviations, it is more difficult to see these regions in the nasal cavities. It is important to stress that the classification was carried out by the three examiners, by means of video recordings and not during the exam itself. This may have made some cases more difficult, thus resulting in different interpretations and then less agreement, especially for the tridimensional staging proposed, which have more categories to be evaluated. It is certain that the agreement for the three methods would have been greater should the exams be made by each examiner. The other staging systems presented better agreement because they are simpler and have fewer categories; however they are less precise in informing the true extension of the polyp. The authors believe that the reproducibility of this new method can be better and will be later reassessed, and it is the one that better describes the true location and extension of the nasal polyp.

## CONCLUSIONS

The staging method hereby proposed proved to be reproducible, despite having less agreement than the other two staging systems.
